# The effect of a high frequency electromagnetic field in the microwave range on red blood cells

**DOI:** 10.1038/s41598-017-11288-9

**Published:** 2017-09-07

**Authors:** The Hong Phong Nguyen, Vy T. H. Pham, Vladimir Baulin, Rodney J. Croft, Russell J. Crawford, Elena P. Ivanova

**Affiliations:** 1 Faculty Science, Engineering and Technology, Swinburne University of Technology, Hawthorn, Vic 3122 Australia; 2 Department d’Enginyeria Quimica, Universitat Rovira I Virgili, 26 Av. dels Paisos Catalans, 43007 Tarragona, Spain; 3 School of Psychology, Illawarra Health & Medical Research Institute, University of Wollongong, Wollongong, NSW, 2522 Australia; 4 Australian Centre for Electromagnetic Bioeffects Research, Wollongong, NSW, 2522 Australia; 5 School of Science, RMIT University, Melbourne, Vic 3001 Australia

## Abstract

The effect of red blood cells (RBC) exposed to an 18 GHz electromagnetic field (EMF) was studied. The results of this study demonstrated for the first time that exposure of RBCs to 18 GHz EMF has the capacity to induce nanospheres uptake in RBCs. The uptake of nanospheres (loading efficiency 96% and 46% for 23.5 and 46.3 nm nanospheres respectively), their presence and locality were confirmed using three independent techniques, namely scanning electron microscopy, confocal laser scanning microscopy and transmission electron microscopy. It appeared that 23.5 nm nanospheres were translocated through the membrane into the cytosol, while the 46.3 nm-nanospheres were mostly translocated through the phospholipid-cholesterol bilayer, with only some of these nanospheres passing the 2D cytoskeleton network. The nanospheres uptake increased by up to 12% with increasing temperature from 33 to 37 °C. The TEM analysis revealed that the nanospheres were engulfed by the cell membrane itself, and then translocated into the cytosol. It is believed that EMF-induced rotating water dipoles caused disturbance of the membrane, initiating its deformation and result in an enhanced degree of membrane trafficking via a quasi-exocytosis process.

## Introduction

The effects of electromagnetic fields (EMFs) upon genes^1–5^, proteins and enzyme kinetics^6–10^ on a molecular level have been recognised and investigated. The mechanisms responsible for these EMF-induced effects and not fully understood and have been the subject of debate^4, 6, 8, 11–15^. It is thought that the effects of EMF are diverse and dependent on the strength, frequency, and duration of the EMF exposures^15, 16^. The EMF microwave effects in GHz frequencies have been studied recently and it was reported that multiple 18 GHz EMF exposures, with specific energy absorption rate (SAR) values between approximately 3.0 and 5.0 kW kg^−1^, induced permeabilization of live bacterial cells and yeast. The uptake of high molecular weight dextran (150 kDa) and silica nanoparticles (23.5 and 46.3 nm in diameter) was shown for several cell types, including the prokaryotic organisms *Branhamella catarrhalis, Escherichia coli, Kocuria rosea, Planococcus maritimus, Staphylococcus aureus*, *Staphylococcus epidermidis*, *Streptomyces griseus*, and a unicellular eukaryotic yeast *Saccharomyces cerevisiae* ^14, 17, 18^.

In light of these findings, the aim of this research was to determine whether exposure to 18 GHz EMF induces cell permeability in red blood cells (RBCs). Gaining an understanding of whether EMF-induced permeability of RBCs can be achieved is of particular interest in the context of RBC preservation, freeze-drying, as well as from a fundamental point of view to induce pinocytosis and endocytosis in RBCs, for RBC-targeted drug delivery platforms^19^. This interest arises because RBCs are biocompatible, biodegradable, non-immunogenic in nature, and less prone to aggregation and fusion^20, 21^. In order to target the reticuloendothelial system or to reduce the extent of allergenic reactions, exogenous materials and/or drugs can be protected from the extracellular environment by encapsulation within RBCs^21, 22^. The plasma membrane of RBCs can protect encapsulated drugs from inactivation with a prolonged and controllable lifespan^20, 21^. Current approaches for the production of RBCs that contain encapsulated drugs exploit the techniques of osmotic diffusion and electroporation due to the minimal effect that these processes have on the structure and morphology of the RBCs^22^. Thus if a degree of cell permeability, similar to that described above for other cell lines, can be achieved in RBCs through 18 GHz EMF exposure, this would offer an additional and potentially important means of drug delivery.

## Results and Discussion

### The morphology of the red blood cells

**Figure 1:**
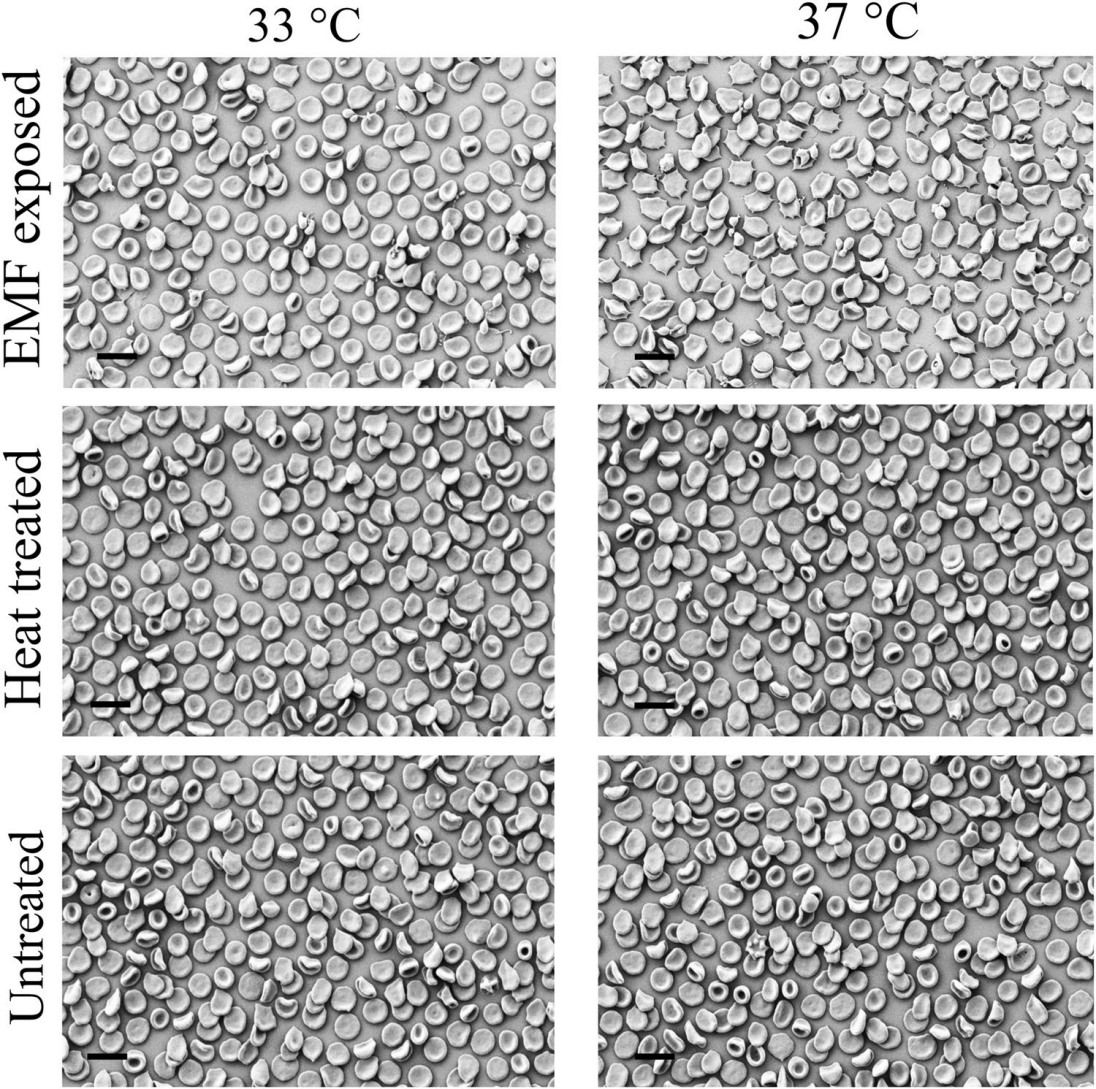
RBC morphology after exposure to an 18 GHz EMF. Typical SEM micrographs of rabbit RBCs after exposure to 18 GHz EMF radiation, at temperatures of up to 33 and 37 °C. Approximately 9% (33 °C) and 14% (37 °C) RBC vesicles (first row) and acanthocytes (first row) were observed. The morphology of the non-treated and Peltier heat-treated control RBCs remained unchanged in their morphology (second and third rows). Scale bars are 10 μm.

The morphology of the RBCs that were subjected to EMF at 33 and 37 °C was examined in order to confirm that the exposure to EMF does not cause physiological death of the RBCs. Analysis of the SEM images showed that the EMF-treated RBCs preserved their original morphology with no statistically significant difference in comparison to the controls (*p* > 0.05). Only 9 ± 1% and 14 ± 1% (at 33 and 37 °C, respectively) of the EMF-exposed RBCs turned into vesicles and acanthocytes, respectively (Fig. 1, top row). According to previous reports, spherisation, partial fragmentation and vesiculization of RBCs could be a result of damaged lipid membrane and/or skeletal proteins such as spectrin^23–25^. Previously the morphological changes in heat-treated human RBCs were recorded at 44 °C^26^, while 49 °C was regarded as the critical temperature for RBC fragmentation^26^. As such, bulk temperature in the range 33 to 37 °C alone, should not affect the morphology of the RBCs. Indeed, it can be seen in Fig. 1 that the morphology of the non-treated and Peltier heat-treated RBCs in control groups remained unchanged (Fig. 1, second and third rows). Hence, the change in the morphology of EMF-exposed RBCs is a manifestation of the change of the area to volume ratio, which in turn, reflects the microscopic changes in membrane elasticity and/or local osmotic pressure. It was also found that approximately 1.5% of the total water present was evaporated from the samples during the EMF exposures, which was considered to be negligible and should not affect the overall osmotic pressure.

### EMF induced nanosphere uptake

**Figure 2:**
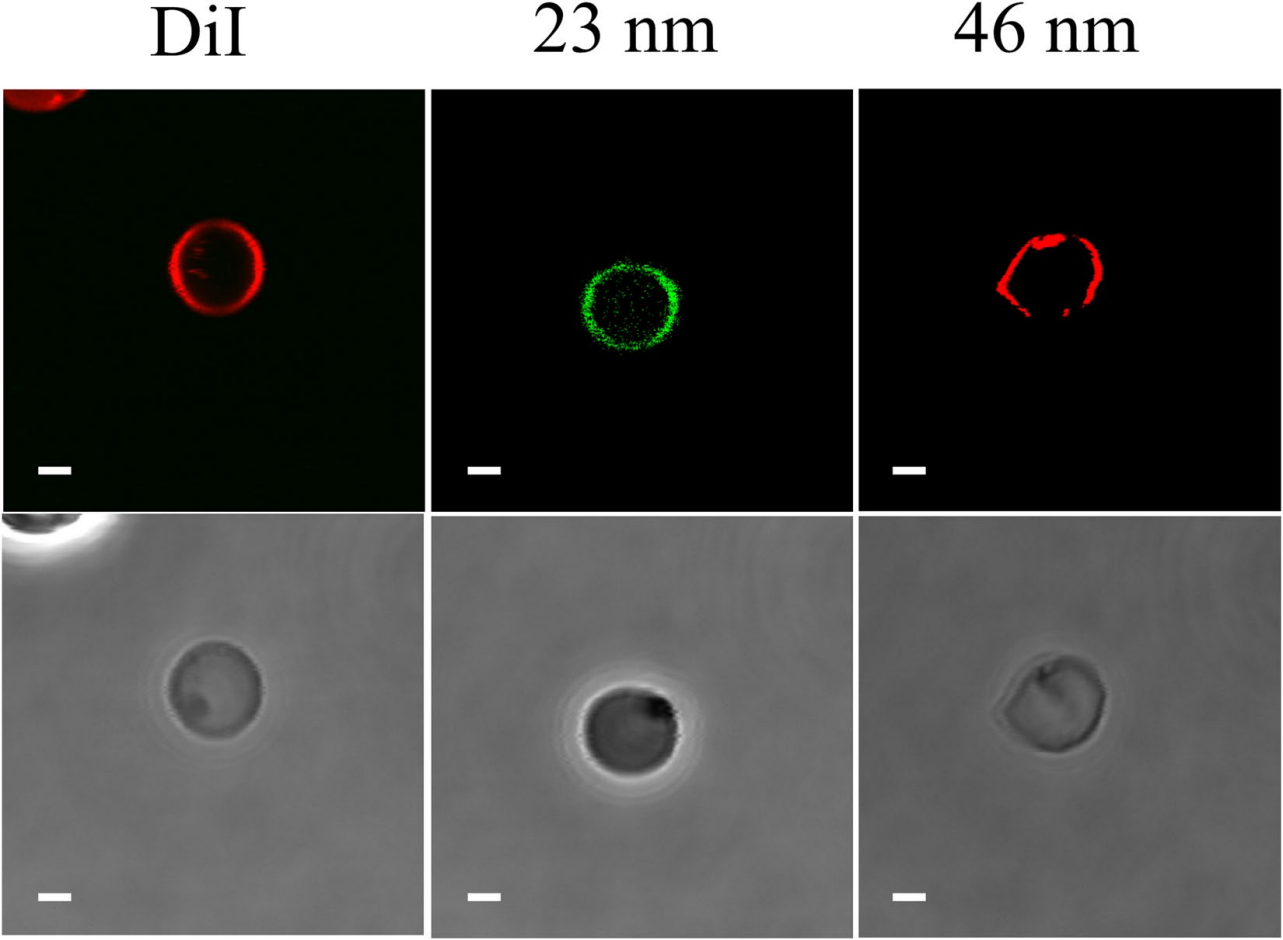
Permeabilization of RBCs resulting from exposure to an 18 GHz EMF. CLSM images show an uptake of 23.5 and 46.3 nm nanospheres (first row). A lipophilic membrane stain, DiI (Life Technologies, Scoresby, VIC, Australia) was used to stain the entire population of RBCs for contrasting purpose (first row). The phase contrast images (second row) show erythrocytes in the same field. Scale bars are 2 μm.

**Figure 3:**
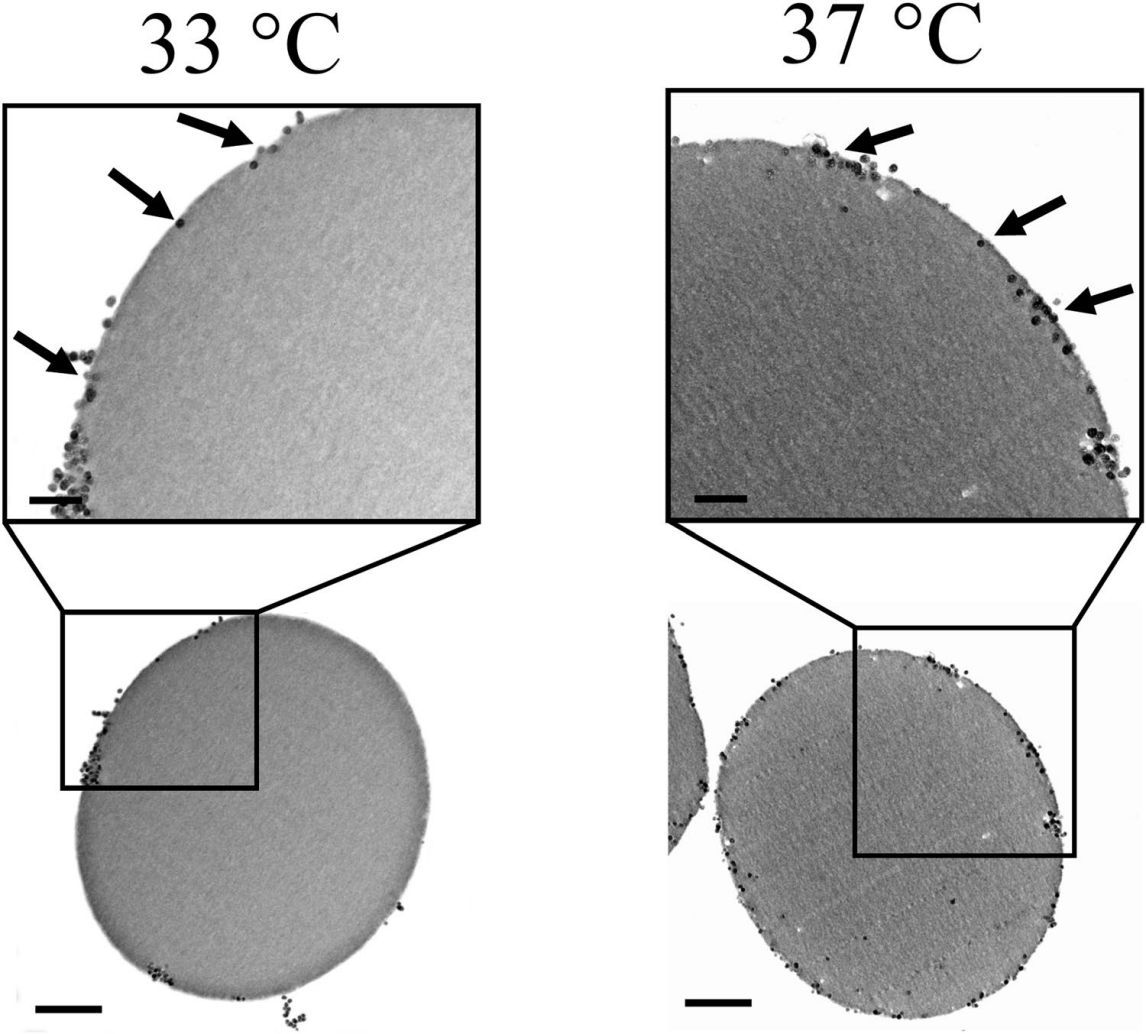
Internalization of 46.3 nm nanospheres into the EMF-exposed RBCs. Typical TEM images of ultra-thin (70 nm) cross-sections of EMF-exposed RBCs, showing the internalization of 46.3 nm nanospheres. The RBCs exposed to EMF and allowed to reach a temperature of 37 °C were able to internalize a greater number of nanospheres than those being exposed to the EMF at a maximum of 33 °C. Scale bars are 0.5 μm (second row). Inset scale bars are 200 nm (first row).

**Figure 4:**
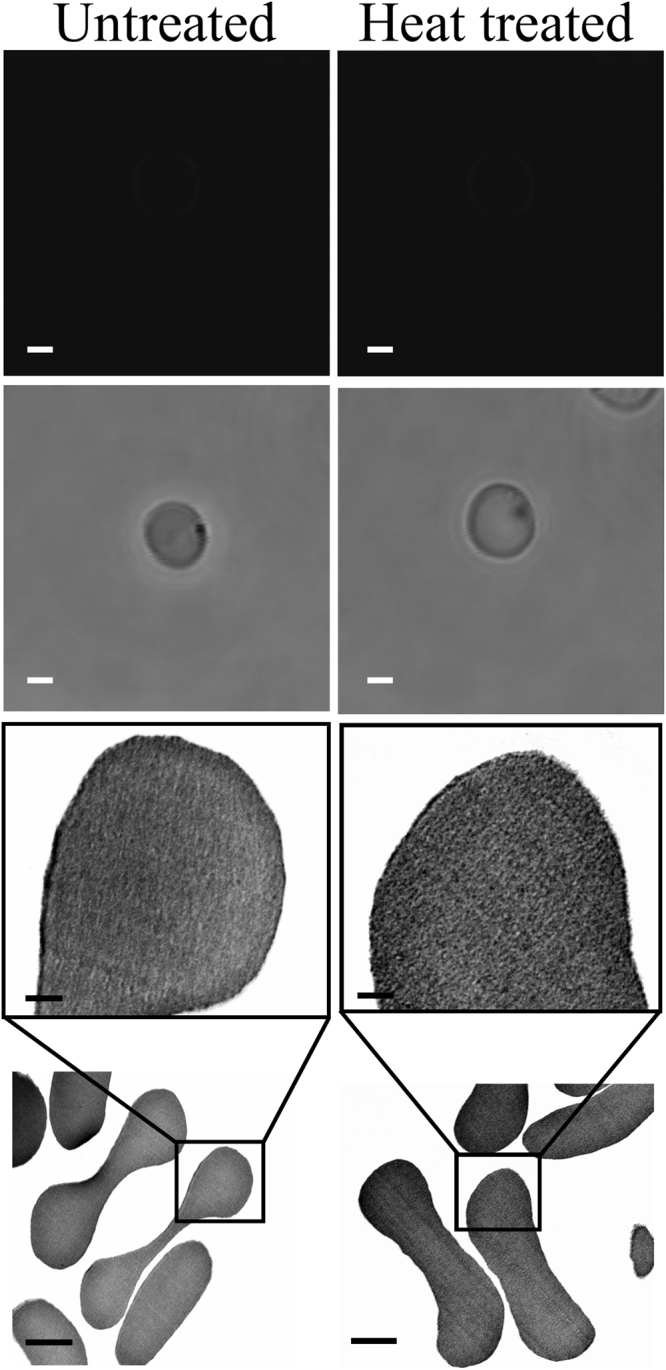
No uptake of 23.5 and 46.3 nm nanospheres by the control groups. CLSM and phase contrast images showing the appearance of the RBCs remaining unchanged, and with no internalization of nanospheres. Scale bars are 2 μm (first and second rows). Typical TEM images of ultra-thin (70 nm) cross-sections RBCs, showing the cell membrane of untreated and heat-treated RBCs with a uniform cytosol without any 46.3 nm nanospheres being present. Scale bars are 1 μm (fourth row). Inset scale bars are 200 nm (third row).

The confocal laser scanning microscopy (CLSM) and transmission electron microscopy (TEM) analysis of RBCs exposed to the EMF showed that the EMF did induce permeability in the membrane of the RBCs, as confirmed by the uptake of silica nanospheres of two different sizes (Figs 2–3). There were statistically significant differences between the EMF exposed RBCs and the control samples (*p* < 0.05). The silica nanospheres were chosen for this study because they are hydrophilic and thus do not cross spontaneously through the lipid bilayer^27^; the sizes of 23.5 nm and 46.3 nm where chosen to be smaller than spectrin mesh size^25, 28^; it has also been reported that the neutrally charged surface of the nanoparticles prevents any nonspecific interactions taking place within the membrane^29^. Thus, this type of nanosphere could be used as the negative control group. The results obtained for both the Peltier heated and untreated RBCs confirmed that in both cases, no nanospheres were taken into the cell membrane (Fig. 4), which rules out bulk temperature change as the cause (there was no statistically significant difference between the EMF and control samples, *p* > 0.05). However, the effect of instantaneous localized temperature elevation (T_i_)^30^ cannot be ruled out as a potential cause. This is because T_i_ can be greater than the bulk temperature (T_B_) in order to satisfy the Arrhenius equation ($k=A{{\rm{e}}}^{-\frac{{E}_{A}}{RT}}$, where E_A_ is the activation energy, R is the gas constant, and T is the temperature)^30^, which means that it is possible that T_i_ differed between the EMF and Peltier plate conditions. This possibility cannot be determined from the present study because T_i_ is a function of EMF energy input and is not directly measurable due to its short existence and molecular nature^30^.

CLSM analysis also indicated that internalization of the nanospheres could continue for up to approximately 9 min after the EMF irradiation (data not shown); whereas no uptake of the nanospheres was detected when the RBCs were exposed to the nanospheres 10 min after the EMF irradiation. This result indicated that the cells remained permeable for 9 min, in agreement with previously reported observations for other cell types^14, 17, 18^.

It was further found here that the loading efficiency of the 23.5 nm nanospheres was over 96% for both 33 and 37 °C temperature conditions with no statistically significant difference (p > 0.05). The loading efficiency of the 46.3 nm nanospheres was, however, 46 and 58% for samples subjected to 33 and 37°C temperatures, respectively (Supplementary Table S1). Overall, the number of RBCs that were able to internalize the 46.3 nm nanospheres increased (up to 12% of total RBCs) when the temperature was increased from 33 to 37°C with a statistically significant difference (p < 0.05) (Supplementary Table S1), which may indicate changes in cellular membrane and spectrin network as a result of increased temperature. Moreover, in the samples that were subjected to the 33 °C and 37 °C temperatures during EMF exposures, a single RBC was estimated to internalize an additional 6 and 10 fg of the 23.5 and 46.3 nm nanospheres, respectively with a statistically significant difference (p < 0.05) (Supplementary Table S1). It should be noted that only the RBCs with circular morphology in the phase contrast images were counted for the quantification of the loading efficiency (Supplementary Fig. S1). The results obtained in this work regarding the temperature-dependent internalization of RBCs were in agreement with the observations reported by Harisa, *et al*.^31^. These authors reported that a greater degree of loading human RBCs with the drug pravastatin occurred at 37 °C compared to that obtained at 25 °C.

A TEM analysis of the ultra-thin (70 nm) cross-sections of the RBCs revealed the intracellular location and the stages of nanosphere translocation. It can be seen from the TEM micrographs that the internalized nanospheres, while being in close proximity to the membrane, appeared to be engulfed by the cell membrane itself, and then translocated into the cytosol (Fig. 3, indicated by arrows). There were also noticeable clusters of nanospheres trapped within the membrane. It was also found, however, that the 23.5 nm-nanospheres were able to cross through the 2D spectrin network into the cytosol (Supplementary Fig. S2, indicated by arrows). The untreated and Peltier plate heated RBCs in the control groups were found not to possess any internalized nanospheres (Fig. 4), highlighting the lack of cell permeability in these samples.

It should be noted that the translocated large nanospheres often remained in close proximity to the membrane on the inside of the RBCs (Fig. 3). The 46.3 nm-nanospheres were most likely trapped between the membrane and the cytoskeletal network, which is located below the RBCs’ membrane. This could be due to the unique organisation of the RBCs’ cell membrane^32^. The asymmetrical phospholipid-cholesterol bilayer, composed of phosphatidylserine, is enriched in the inner leaflet^33^. Underneath the phospholipid-cholesterol bilayer is a two-dimensional (2D) cytoskeleton that is approximately 7.9 nm in thickness^28^. This cytoskeleton network is anchored to the phospholipid bilayer, which has the dimensions of an approximately 162 nm × 65 nm (length × width) mesh^25, 28^. The 2D cytoskeleton consists of spectrin heterodimers and actin bundles and is involved in the process of cell volume regulation^25^. This skeletal network allows RBCs to undergo significant extensional deformation whilst maintaining their structural integrity^34^. The results obtained in this work suggested that while the 46.3 nm-nanospheres were able to be translocated through the phospholipid-cholesterol bilayer, only 23.5 nm nanospheres could pass the 2D cytoskeleton network.

Thus, it was demonstrated that three consecutive 18 GHz EMF exposures, each of 1 min with SAR of approximately 3.0 kW kg^−1^ (at 33 °C), consistently induced membrane permeabilization in RBCs without compromising the viability of the cells. Since the 18 GHz EMF is a rapidly alternating field, it acts directly on the water dipoles, inducing their rotation and reorientation with a polarization lag being responsible for the dissipation of energy and heating^35, 36^. The rotation of water molecules disrupts the hydrogen bonding network and therefore affects, among others, the dielectric constant of the water^35, 36^. The energy dissipation in bulk close to membranes can affect the membrane stability. This change in activity might induce the cell membrane disturbance, rendering the cells more permeable and sensitive to membrane deformation^37–41^. The EMF-induced mechanical disturbance might alter the membrane tension permeability, and thus an enhanced degree of membrane trafficking through lipid bilayer. The membrane remained permeable for approximately nine minutes then was restored to its original state after 10 minutes, as has been confirmed for different cell types^14, 17, 18^. This may be due to long relaxation processes in lipid membranes after mechanical disturbance. It has been reported that RBCs may undergo invagination of their cell membrane, which can then be ‘pinched off’ and sealed, forming intracellular vacuoles^42^. This internalization of the cell membrane is reminiscent of the pinocytosis that has been observed in other cell types^42^. Ginn *et al*. suggested that this invagination process would result in a decrease in surface area of cell membranes under tension, thus reducing the critical haemolytic volume of the cell^42^. The consequences of membrane internalization are, therefore, similar to those produced by fragmentation, in that both processes will result in a decrease in the overall area of the cell surface membrane^42^.

The results of this study have demonstrated, for the first time, that exposing RBCs to 18 GHz EMF has the capacity to induce cell permeability without compromising cell viability. This effect was not found in the Peltier heating control condition, which shows that it was not due to bulk temperature rise, however, whether more spatially and temporally localised temperature changes may account for the effect cannot be determined from the present study. Upon EMF exposure, the RBC membranes are thought to become permeable due to mechanical membrane disturbance. We believe that the EMF-induced nanoparticle uptake is a unique and universal phenomenon, because diverse cell types could be equally affected in a similar way. The elucidation of the molecular mechanism/s of the EMF-induced permeability will require further investigation.

## Material and Methods

### Silica nanospheres

Two types of fluorescent silica nanospheres, 23.5 ± 0.2 nm (FITC) and 46.3 ± 0.2 nm (Rhodamine B) in diameter, purchased from Corpuscular, Cold Spring, NY, USA.

### Isolation of RBCs

RBCs were isolated from the fresh blood of a 16 week-old New Zealand white rabbit according to the method approved by the Monash Animal Ethics Committee (Physiology, Monash University, Australia). The whole blood was placed into plastic Vacutainer tubes, spray-coated with K_2_EDTA (Becton Dickinson, Sparks, NV, USA) and inverted several times to prevent blood clotting. The Vacutainer tubes were then disinfected using 70% ethanol. All experimental procedures were carried out within a class II bio-safety cabinet (Thermo Fisher Scientific, Waltham, Massachusetts, USA) according to the approval obtained from the Swinburne Biosafety Committee (Swinburne Research, Swinburne University of Technology, Australia). The fresh blood was then gently homogenized with a 10 mM phosphate buffer saline (PBS) solution at pH 7.4 and at a 1:1 ratio. In a Falcon tube (Becton Dickinson), the blood mixture was then carefully placed over a Histopaque 1077 solution (Sigma Aldrich, St. Louis, Missouri, USA) at a 3:1 ratio, then centrifuged at 400 g for 20 min. The layer of RBCs at the bottom of the Falcon tube was recovered and washed twice in a 1.0 mM PBS solution at pH 7.4, then centrifuged at 400 g for 5 min. The cell density was then adjusted to 5 × 10^5^ cells mL^−1^ in PBS using a Neubauer-improved haemocytometer (Paul Marienfeld, Lauda-Königshofen, Germany). This concentration allowed the EMF energy to be evenly distributed amongst RBCs and the specific heat capacity of the cell suspension was approximately equivalent to that of water at 25 °C.

### Dosimetry

The dose of EMF that was delivered to the RBC samples is expressed as the specific absorption rate (SAR, kW/kg), as described elsewhere^17, 18, 43^. In brief, the SAR was measured under the assumption that all of the absorbed field energy was transformed into heat, with any heat dissipation being disregarded, according to:

1 $$SAR=c\times {\frac{\partial T}{\partial t}|}_{t=0}$$

where *c* is the specific heat capacity of the medium (kJ kg^−1^ °C^−1^), and ${\frac{\partial T}{\partial t}|}_{t=0}$ is the time derivative of the temperature, determined at t = 0 s (°C s^−1^).

The SAR of each EMF exposure was calculated using Equation (1) by measuring the temperature change over time under the optimum settings (power, temperature and number of treatments). It was assumed that the specific heat capacity of the cell suspension was equivalent to that of water at 25 °C, which is 4.18 kJ kg^−1^ °C^−1^ ^17, 18^. Any liquid evaporation that took place during the EMF exposures was measured in triplicate after each of the EMF exposures. The samples were weighed before and after EMF exposures to determine the amount of evaporated water using an analytical balance (Cheetah Scientific, France).

### EMF treatment

**Figure 5:**
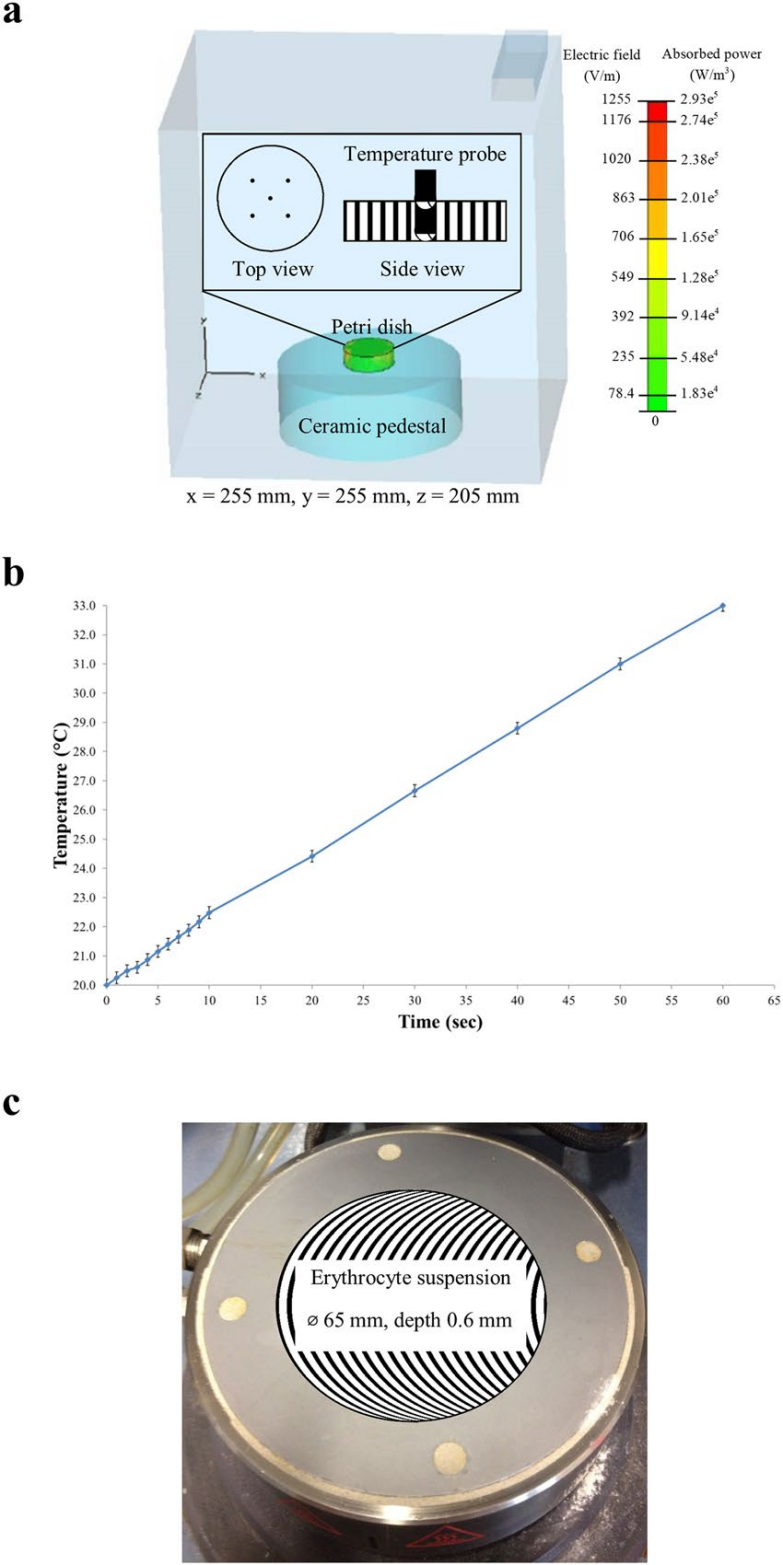
The energy distribution and the exposure system of the 18 GHz EMF. (**a**) Electric field and absorbed power modeling using CST Microwave Studio 3D Electromagnetic Simulation Software and inset images of the tip positions of the temperature probe in media (top and side view). (**b**) The heating rate of the RBCs suspension during EMF exposure.

RBC samples were subjected to EMF exposures following the procedures as described elsewhere^14, 17, 18^. In brief, 2 mL of the RBC suspensions were subjected to three consecutive EMF exposures of approximately 3.0 kW kg^−1^ SAR per exposure. The EMF apparatus used for all experiments was a Vari-Wave Model LT 1500 (Lambda Technologies, Morrisville, NC, USA) instrument with a fixed frequency of 18 GHz. The samples were placed onto a ceramic pedestal PD160 (Pacific Ceramics, Sunnyvale, CA, USA, ε′ = 160, loss tangent <10^−3^) at a position that had been identified, using electric field modelling of CST Microwave Studio 3D Electromagnetic Simulation Software (CST MWS) (CST of America, Framingham, MA, USA), to be the position that provided the most consistent heating environment (Fig. 5a). The calculated wavelength of the EMF in water was determined to be 2.34 mm, which is greater than the linear dimensions of each bacterial cell. The depth of penetration was calculated to be 1.04 mm, which was greater than the thickness of the RBCs suspension in the Petri dish. Hence, the possibility of subjecting the samples to non-even heating due to the presence of a non-uniform field distribution was considered to be negligible. The temperature of the suspension was constantly monitored during EMF exposures via a built-in temperature probe, a Luxtron Fiber Optic Temperature Unit (LFOTU) (LumaSense Technologies, Santa Clara, CA, USA), and a portable Cyclopes 330 S infrared/thermal monitoring camera (Minolta, Osaka, Japan). The EMF exposures resulted in a temperature increase in the sample that ranged from 20 to 37 °C (at a heating rate of 13 °C per min). After exposure, samples were allowed to cool to 20 °C on ice (at a rate of 10 °C per min) before any subsequent exposures were administered. In order to minimise the morphological changed RBCs to less than 10% of total RBCs, the EMF maximum exposure temperature was then decreased to 33 °C (at a heating rate of 13 °C per min). After a temperature in the sample reached 33 °C, the samples were allowed to cool to 20 °C on ice (at a rate of 10 °C per min) between exposures. The heating rate arising from the EMF exposure is shown in Fig. 5b. Every EMF optimization step was validated by performing up to five independent experiments, each performed in triplicate, to allow a statistical analysis of the results to be performed.

### Bulk heat treatment

A Peltier plate heating/cooling system (TA Instruments, New Castle, DE, USA) was used to replicate the bulk temperature profiles experienced by the RBC samples during the EMF exposure experiments, the method of which is described elsewhere^14, 17, 18^. Briefly, a 2 mL volume of RBCs suspension was applied directly onto the Peltier plate sample platform (Fig. 5c), and subjected to the same bulk temperature profiles experienced by the RBC samples during the EMF exposure experiments. The diameter of the Peltier plate sample platform was 65 mm and the RBCs suspension layer thickness was calculated to be 0.6 mm. All Peltier plate heated samples were studied in parallel with the EMF exposure experiments, with at least three independent experiments being conducted. Working RBC suspensions that were not subjected to either EMF exposure or Peltier plate heating were used as negative controls (performed in triplicate for each independent experiment) for all experiments.

### Confocal Laser Scanning Microscopy

A lipophilic membrane stain, DiI (Life Technologies, Scoresby, VIC, Australia) was used to stain the RBC samples for contrasting. Before commencing the EMF exposure or heat treatment, DiI was added to the samples at a concentration of 5 µL per mL of cell suspension, allowed to incubate for 20 min at 37 °C, and then washed twice by centrifugation at 1300 rpm for 5 min.

After EMF exposures or heat treatment, the two different diameter nanospheres were added to the cell suspensions at a concentration of 50 µg mL^−1^, allowed to incubate for 10 min, then washed twice using centrifugation at 1300 rpm for 5 min. A 100 µL aliquot of each sample was then analyzed using a Fluoview FV10i-W inverted microscope (Olympus, Tokyo, Japan). Approximately 10 CLSM images per EMF exposure group were obtained for a subsequent statistical analysis. For each CLSM image, the number of morphologically unchanged RBCs was manually counted.

### Quantification of cell permeability

The nanosphere loading capacity of the EMF-exposed RBC samples was quantified using the fluorescence intensity of the silica nanospheres that were internalized by the cells using a POLARstar Omega microplate reader (BMG Labtech, Ortenberg, Germany). Each sample was prepared according to the method used for CLSM analysis. A calibration curve was constructed to determine the correlation of the fluorescent intensity with the concentration of nanospheres. A total of eleven concentrations of nanospheres were prepared (0.005, 0.006, 0.007, 0.008, 0.009, 0.01, 0.011, 0.012, 0.013, 0.014 and 0.015 µg mL^−1^).

The mass *m* of a silica nanosphere was determined from the density of silica *ρ* and the volume of a silica nanosphere *V*, related to the radius *r* as $V=\frac{4}{3}\pi {r}^{3}$.

The radii of the two nanosphere types were 11.75 × 10^−7^ cm and 23.15 × 10^−7^ cm, (Corpuscular), and hence their volumes were 6.8 × 10^−18^ and 5.2 × 10^−17^ cm^3^, and mass 1.8 × 10^−17^ and 1.38 × 10^−16^ g, respectively. The mass of a single nanosphere was used to calculate the number of internalized nanospheres.

All fluorescence intensity measurements were performed in up to five independent experiments, each in triplicate to allow a subsequent statistical analysis.

### Scanning Electron Microscopy

A field emission scanning electron microscope FeSEM – SUPRA 40VP (Carl Zeiss, Jena, Germany), with a primary beam energy of 3 kV, was used to obtain high-resolution images of the cell samples. A 100 µL aliquot of each sample was placed on a glass cover slip (ProSciTech, Kirwan, Australia), in duplicate, and allowed to sit for 10 min. All samples were then fixed in 2.5% glutaraldehyde (Sigma) for 30 min and progressively dehydrated using graded ethanol solutions (30, 50, 70, 90, and 100% v/v) for 10 min. The glass cover slips were air-dried and then subjected to gold sputtering (6 nm thick gold film) using a NeoCoater MP-19020NCTR (JEOL, Frenchs Forest, Australia) instrument. Approximately thirty SEM images for each experimental step were obtained at a magnification of 3,000× for subsequent statistical analysis. For each SEM image, the number of morphologically unchanged RBCs was manually counted.

### Transmission Electron Microscopy

The 46.3 nm nanospheres were added to the cell suspensions after they had been subjected to the EMF exposures, and then pelleted using centrifugation at 1300 rpm for 5 min at 25 °C. The cells were then washed twice with 10 mM PBS at a pH of 7.4 in order to remove any nanospheres that had not been internalized by the cells. The resulting pellets were then suspended in 2 mL of 1% glutaraldehyde in PBS for 30 min, and then washed twice in PBS for 5 min. After the final washing step, the cell suspensions were added to 0.5 mL of molten 3% agarose gel by piercing the gel with the tip of the pipette. The agar was then immediately cooled to 4 °C by refrigeration for 30 min, and then the area containing the cell suspension was trimmed from the total agar area. Samples containing the cell suspension were washed twice in nanopure H_2_O (with a resistivity of 18.2 MW cm^−1^) for 15 min each. Samples were then dehydrated by passing them through a graded ethanol series (20, 40, and 60%) (2 mL) for 15 min and then stained for 8 h with 2% uranyl acetate in 70% ethanol (2 mL). After staining, the cells were further dehydrated by passing the samples through another graded ethanol series (80, 90 and 100%) for 15 min each (2 mL).

The embedding medium was prepared using LR gold resin (ProSciTech). In order to embed the samples, each agar sample containing the cell suspension was incubated in 2 mL of 100% ethanol and LR gold monomer (1:1 ratio) for 8 h, followed by a transfer to 100% ethanol and LR gold monomer (1:3 ratio) for 8 h, then finally a transfer into the pure LR gold monomer for 8 h. Each sample was then transferred into a gelatin capsule containing fresh LR gold monomer mixed with 1% dry benzoyl peroxide, which was then polymerized for 24 h at 4 °C. The final block was trimmed, then cut into ultrathin sections (70 nm thickness) using a Leica EM UC7 Ultramicrotome (Leica Microsystems, Wetzlar, Germany) with a diamond knife (Diatome, Pennsylvania, USA). Sections were placed onto 200 mesh copper grids and examined using a JEM 1010 instrument (JEOL). Approximately 40 TEM images were taken at × 5000 and × 10000 magnifications for each sample analysed.

### Statistical analysis

All statistical data processing was performed using SPSS 22.0 software (SPSS, Chicago, IL, USA).

## Electronic supplementary material

Supplementary info
