# Supplementary material for: The effect of a high frequency electromagnetic field in the microwave range on red blood cells
Source: Sci Rep. 2017 Sep 7;7:10798. doi: 10.1038/s41598-017-11288-9 (PMC5589725; doi:10.1038/s41598-017-11288-9)

The effect of a high frequency electromagnetic field in the microwave range on red blood cells

The Hong Phong Nguyen[1], Vy T. H. Pham[1], Vladimir Baulin[2], Rodney J. Croft[3,4], Russell J. Crawford[5] and Elena P. Ivanova[1,4]*

[1] Faculty Science, Engineering and Technology, Swinburne University of Technology, Hawthorn, Vic 3122, Australia

[2] Department d'Enginyeria Quimica, Universitat Rovira I Virgili, 26 Av. dels Paisos Catalans, 43007 Tarragona, Spain

[3] School of Psychology, University of Wollongong, Wollongong, NSW 2522, Australia

[4]Australian Centre for Electromagnetic Bioeffects Research, Australia

[5] School of Science, RMIT University, Melbourne, Vic 3001, Australia

*Corresponding author mailing address: School of Science, Swinburne University of Technology, PO Box 218, Hawthorn, Vic 3122, Australia; Telephone: +61392145137; Facsimile: +61398190834; e-mail: eivanova@swin.edu.au

Supplementary Table S1. Internalization of silica nanospheres by RBCs subjected to EMF irradiation.

	Silica nanospheres			
	23.5 nm		46.3 nm	
**Temperature (°C)**	**Loading capacity (fg)***	**Loading efficiency (%)**	**Loading capacity (fg)***	**Loading efficiency (%)**
33	16 ± 1	96 ± 5	14 ± 1	46 ± 6
37	22 ± 1	98 ± 5	24 ± 1	58 ± 6

* per single RBC

The nanosphere loading capacity was calculated using the fluorescence intensity of the nanospheres. The number of RBCs that were able to internalize the nanospheres, expressed as a percentage, was calculated by counting the total number of fluorescent cells in the CLSM images. Data are mean ± standard deviation, and are representative of 3 independent experiments.

SUPPLEMENTARY FIG. S1| Quantification of permeabilized RBCs, resulting from exposure to an 18 GHz EMF. CLSM images show an uptake of 23.5 and 46.3 nm nanospheres (first row). The phase contrast images (second row) show RBCs in the same field. Only the RBCs with circular morphology in the phase contrast images were counted for the quantification.   Scale bars are 10 μm.

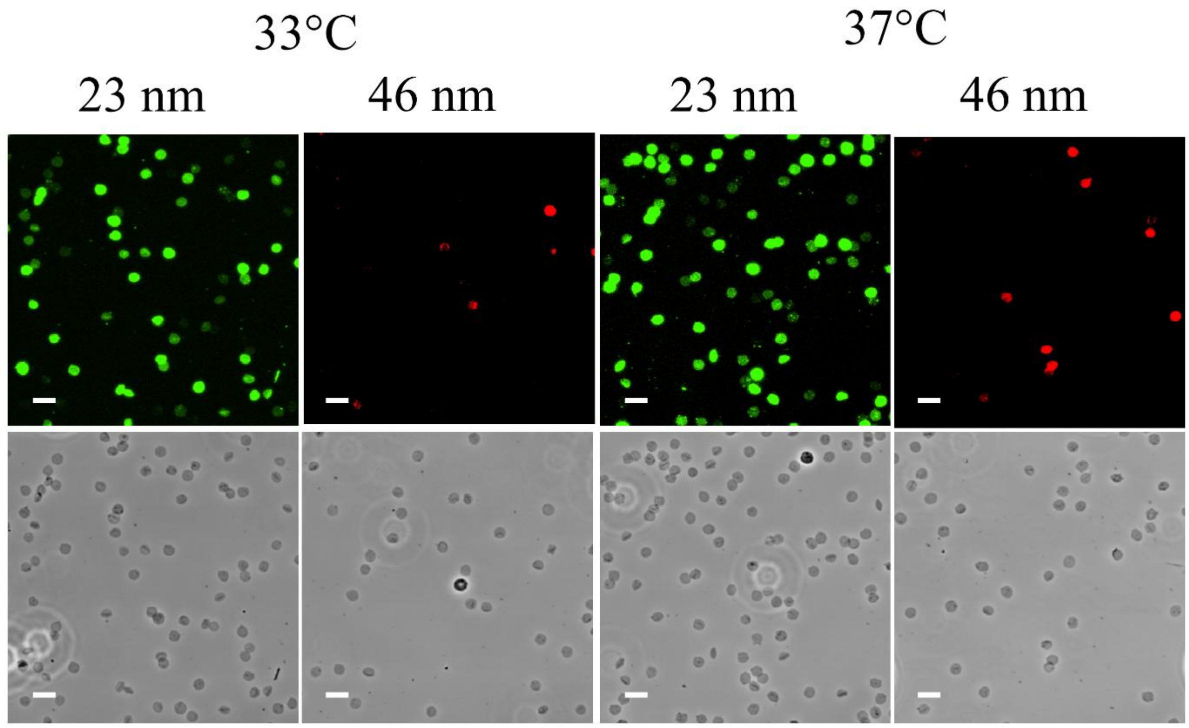

SUPPLEMENTARY FIG. S2| Internalization of 23.5 nm nanospheres into the EMF-exposed RBCs. Typical TEM images of ultra-thin (70 nm) cross-sections of EMF-exposed RBCs, showing the internalization of 23.5 nm nanospheres. It appeared that the 23.5 nm nanospheres were able to crossover the 2D spectrin network into the cytosol (indicated by arrows). Scale bars are 200 nm.

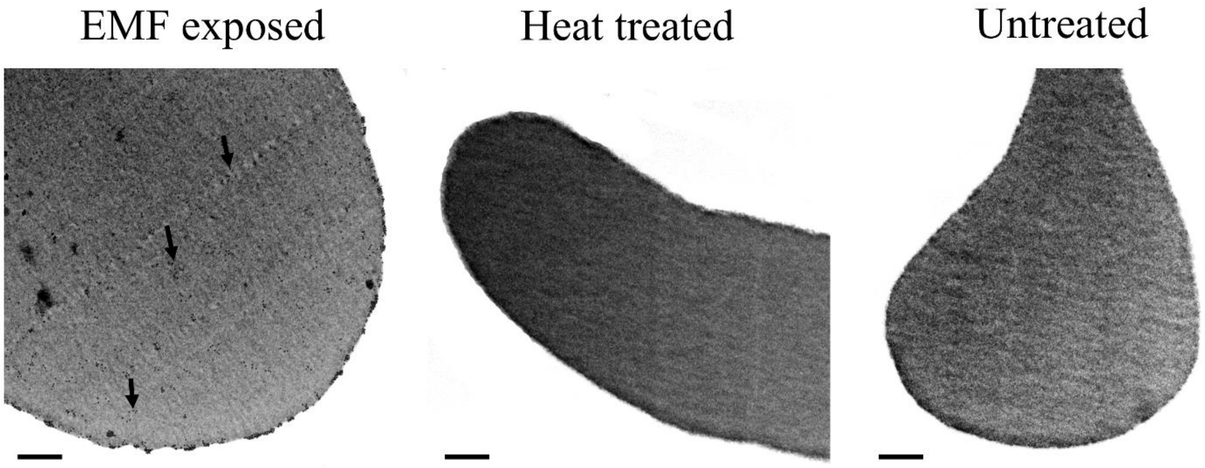

Supplement: Supplementary file 1 — Supplementary info [file 41598_2017_11288_MOESM1_ESM.pdf]
